# Are We Overdoing It? Changes in Diagnostic Imaging Workload during the Years 2010–2020 including the Impact of the SARS-CoV-2 Pandemic

**DOI:** 10.3390/healthcare9111557

**Published:** 2021-11-16

**Authors:** Mateusz Winder, Aleksander Jerzy Owczarek, Jerzy Chudek, Joanna Pilch-Kowalczyk, Jan Baron

**Affiliations:** 1 Department of Radiology and Nuclear Medicine, Medical University of Silesia, 40-055 Katowice, Poland; jpk@alteris.pl (J.P.-K.); janb@onet.pl (J.B.); 2 Health Promotion and Obesity Management Unit, Department of Pathophysiology, Medical University of Silesia, 40-055 Katowice, Poland; aowczarek@sum.edu.pl; 3 Department of Internal Medicine and Oncological Chemotherapy, Medical University of Silesia, 40-055 Katowice, Poland; chj@poczta.fm

**Keywords:** diagnostic imaging, radiology, workload, COVID-19

## Abstract

Since the 1990s, there has been a significant increase in the number of imaging examinations as well as a related increase in the healthcare expenditure and the exposure of the population to X-rays. This study aimed to analyze the workload trends in radiology during the last decade, including the impact of COVID-19 in a single university hospital in Poland and to identify possible solutions to the challenges that radiology could face in the future. We compared the annual amount of computed tomography (CT), radiography (X-ray), and ultrasound (US) examinations performed between the years 2010 and 2020 and analyzed the changes in the number of practicing radiologists in Poland. The mean number of patients treated in our hospital was 60,727 per year. During the last decade, the number of CT and US examinations nearly doubled (from 87.4 to 155.7 and from 52.1 to 86.5 per 1000 patients in 2010 and 2020 respectively), while X-ray examinations decreased from 115.1 to 96.9 per 1000 patients. The SARS-CoV-2 pandemic did not change the workload trends as more chest examinations were performed. AI, which contributed to the COVID-19 diagnosis, could aid radiologists in the future with the growing workload by increasing the efficiency of radiology departments as well as by potentially minimizing the related costs.

## 1. Introduction

From the very beginning, when William Roentgen discovered X-radiation in 1895, radiology has been booming. Further work on X-rays and subsequent discoveries have led to the emergence of computed tomography (CT) and other modalities such as ultrasonography (US) or magnetic resonance imaging (MRI) that changed the perception of human bodies, diseases, and their diagnoses. Even though no new imaging techniques have been invented, the current ones are continuously evolving and improving utilizing technology innovations. Modern radiological modalities are characterized by higher imaging resolution and shorter scanning times, allowing for more and better quality examinations to be performed.

In recent years, diagnostic imaging facilities have been growing in numbers and have become more modern as the demand for radiological examinations has increased [1,2,3]. Imaging not only facilitates the diagnosis of numerous pathologies and the effects of surgical procedures but is also an obligatory part of modern drug programs–especially in systemic cancer treatment [4]. However, with the benefits of greater availability of imaging studies, the risk of population-based exposure to X-rays increases. In addition, X-ray imaging techniques are also widely used in cardiology and interventional neurology, radiotherapy, and other specialties besides radiology. It is estimated that the background and natural radiation is 1–3 mSv per year whereas an average radiation dose for a single CT scan is 10 to 30 mSv [5,6]. Studies show that the risk of cancer increases with radiation exposure, especially in children, and medical procedures using radiation are responsible for 0.6% to 3% of cancers worldwide [7,8,9]. In order to limit the exposure of patients to medical radiation, a consensus called “As Low as Reasonably Achievable” (ALARA) has been introduced into radiological practice, which seems to be justified beyond doubt [10].

The increasing quantity of diagnostic imaging examinations performed has been the subject of some studies, but they have drawn extremely important conclusions regarding the population’s growing exposure to radiation and the costs of imaging services [1,2,11]. A study conducted in the USA analyzing imaging examinations performed in the years between 1997 to 2006 showed that the number of CT scans performed during that time and the costs associated with all imaging modalities (US, CT, MRI) had doubled [1]. The key question is how healthcare is dealing with the almost logarithmic increase in the number of examinations and what actions should be taken to deal with the inevitable upcoming challenges resulting from this fact.

Healthcare expenditure in Europe, based on a percentage of gross domestic product (GDP), ranges from 3.7% in Lithuania to 9.7% in Germany (4.5% in Poland and 14.3% in the USA) [12,13]. In 2012 in Italy, 36% of healthcare expenses in specialist clinics went to imaging tests, surpassing other services including laboratory diagnostics and therapeutics [3].

A rapidly growing number of imaging techniques translates into an excessive workload, which is one of the major risk factors responsible for the occurrence of burnout among physicians and contributes to more mistakes being made [14,15,16]. The data provided by Eurostat shows a large disproportion in the physicians to inhabitants ratios in the European Union (EU) Member States. The number of practicing physicians per 100,000 inhabitants varies from 238 in Poland (2017) to 610 in Greece (2018) [12]. The shortage of doctors experienced in many countries is, itself, conducive to a higher workload.

Recent epidemiological events are extremely interesting in terms of diagnostic imaging, especially due to the possible impact on the progress of radiology in the future. The onset of the SARS-CoV-2 pandemic in 2020 has resulted in unprecedented changes in the functioning of healthcare systems across the world. Healthcare professionals and government officials faced new challenges related to the development of novel COVID-19 treatment, effective prophylaxis, and sensitive diagnostics of the infection and its consequences. In this respect, imaging examinations, mainly high-resolution chest CT (HRCT), have proved to be the main diagnostic tool. The recommendations of radiological societies state that the chest CT should be used sparingly and reserved for hospitalized, symptomatic COVID-19 patients with specific clinical indications for CT [17,18].

The rapid increase in the number of lung examinations that followed the outbreak of COVID-19 inspired the creation of algorithms that would analyze the data acquired from radiological examinations in order to improve and simplify this diagnosis [19,20]. The idea behind these programs originated from a concept of radiomics—a mathematical method of analyzing the medical imaging data to enhance its interpretation [21].

The need for fast adaptation in the healthcare industry during the SARS-CoV-2 pandemic, as well as fear-driven delaying of medical care by the patient, resulted in a periodic decrease in the number of elective imaging examinations performed [22,23]. It seems, however, that the growing, pre-pandemic trend in the quantity of diagnostic imaging is back on track.

This study presents the analysis of the workload trends in radiology during the last decade, including the impact of COVID-19, in a single university hospital, and aims to identify possible solutions to the challenges that radiology might face in the near future.

## 2. Materials and Methods

### 2.1. Materials

For the study, we compared the annual amount of diagnostic imaging examinations and interventional radiology procedures performed between the years 2010 and 2020 in a single-center, the largest hospital in Upper Silesia voivodship in Poland—the University Clinical Center (UCC) of the Medical University of Silesia in Katowice. All the analyzed examinations were performed by the radiologists working in the Department of Radiodiagnostics, Interventional Radiology and Nuclear Medicine of the aforementioned hospital.

The radiographic (X-ray), US, CT examinations, and interventional radiology procedures performed during each year between 2010 and 2020 were summed up. MRI examinations were not included in the analysis as they were performed by an external company providing services to the hospital and mainly the private sector.

Additional analysis of the chest CT examinations from 2019 and 2020 was performed and included HRCT or chest CT with and without intravenous (iv) contrast administration as well as CT examinations of the chest performed alone or together with the surrounding anatomical areas due to other diseases, mainly oncological ones.

In 2020, during the SARS-CoV-2 pandemic, chest CT examinations were performed in (1) symptomatic COVID-19 positive (+) patients (assessment of the degree of the disease), (2) antigen and/or RT-PCR negative (−) but symptomatic patients suspected of having coronavirus infection, and (3) patients admitted to the hospital for other urgent reasons and suspected of having coronavirus infection.

The number of performed examinations and procedures was calculated per 1000 adult patients who were admitted to the emergency room (ER), other hospital departments, hospital clinics as well as referred to the Department of Radiology from external clinics.

### 2.2. Data Analysis

The data regarding the number of patients receiving medical services was officially provided by the Department of Contracting and Billing of Benefits of the UCC hospital. No personal data were acquired or analyzed.

The mean annual number of patients treated was 60,727. The highest annual number of patients reached 67,555 in 2010 and the lowest annual number of patients was observed in 2020 (52,316) due to the SARS-CoV-2 pandemic.

The complete data are presented in Table 1.

The data regarding the number of practicing radiologists in Poland in 2010 and 2020 was officially provided by the Polish Chamber of Physicians and Dentists. Additional data was acquired from the official Statistics Poland and Eurostat publications.

### 2.3. Analysis of the Hospital Organization Changes

In 2015 the number of beds in the Intensive Care Unit was increased by two from 8 to 10 in total.

In 2018 the pneumology department was closed and replaced by the allergology department.

Since April 2020 the Internal Medicine 1 and Stroke Departments have been transformed into specialized COVID-19 units. The rest of the UCC departments have been partially transformed or remained COVID-free (Allergology, Neonatology, Neurological Rehabilitation).

Apart from that, no other significant changes occurred regarding the functioning of the Hospital.

### 2.4. Statistical Analysis

Statistical analysis was performed using STATISTICA 13.0 PL (TIBCO Software Inc., Palo Alto, CA, USA). Data were presented as numbers and percentages as well as incidence rate per 1000 patients. Time trends were presented, and the linear regression model was used to assess the increase in bedside X-rays incidence rate through time. Nominal and ordinal data were compared with the chi-squared test. Statistical significance was set at a *p*-value below 0.05.

## 3. Results

**Figure 1:**
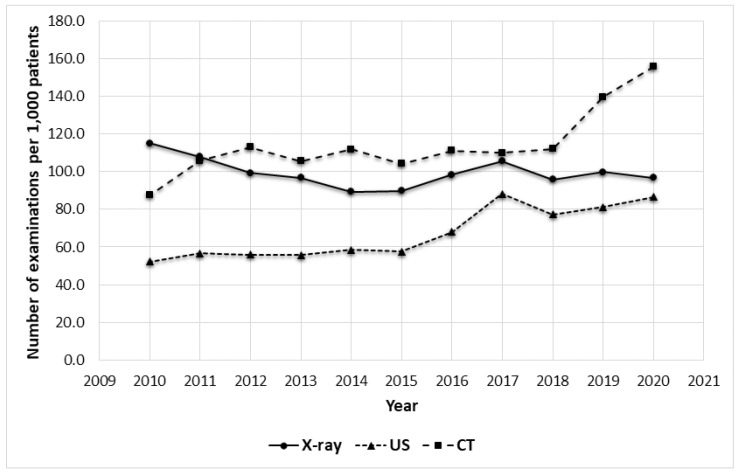
Annual changes in the number of CT, US, and X-ray examinations.

During the last decade (2010–2020), the number of CT examinations increased by 1.8 times, from 87.4 to 155.7 per 1000 patients. Although the overall increase reached 78%, the annual number of CT scans between the years 2011 and 2018 differed insignificantly and accounted for around 109 per 1000 patients. A rapid increase was noted in the following two years (2019 and 2020) when the number of CT examinations increased by 1.25 and 1.4 times (25% and 39% increase) compared to 2018, respectively (Figure 1). This steady trend has not been changed by the SARS-CoV-2 pandemic, despite the much smaller number of patients treated that year. The reason for this was a significant increase in the chest CT examinations performed in 2020 that resulted from pandemic lung imaging recommendations (Table 2) [17,18].

Compared to the previous year, in 2020 there was a 4.4 fold increase in the number of HRCT examinations (from 2.0 to 8.7 per 1000 patients), 3.0 fold increase in HRCT and chest CT examinations together (from 9.5 to 28.6 per 1000 patients), and 4.9 fold increase in any CT examinations that included the chest area (from 10.8 to 52.3 per 1000 patients).

US examinations also increased in number by 1.7 times, from 52.1 to 86.5 per 1000 patients in 2010 and 2020 respectively (66% increase).

**Figure 2:**
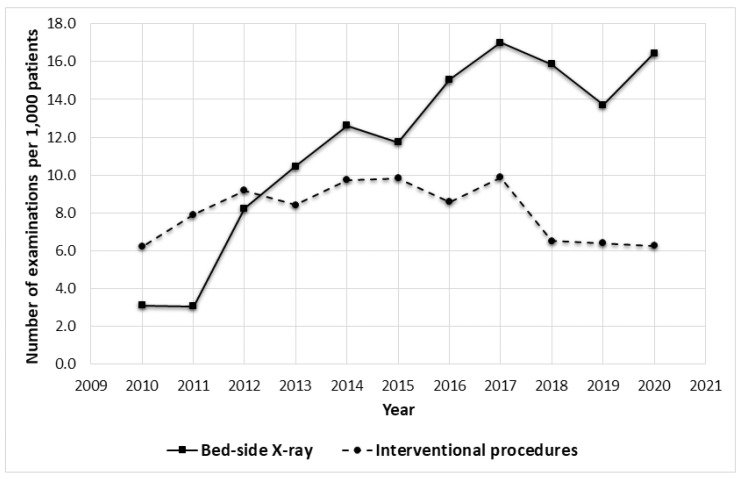
Annual changes in the number of bedside X-ray examinations and interventional procedures.

Notwithstanding, the number of X-ray examinations, of which the vast majority were chest radiographs, decreased from 115.1 to 96.9 per 1000 patients (16% decrease). Yet the overall level through the decade was around 100 per 1000 patients and the lowest number—89.3 per 1000 patients was noted in 2014. However, the number of bedside X-rays increased dramatically by 5.3 times from 3.1 in 2010 to 16.4 per 1000 patients in 2020, reaching the highest peak (17.0) in 2017 (Figure 2). Each year resulted in 30% increase of this type of examination (linear trend, r = 0.90; *p* < 0.001).

Interventional radiology procedures showed an increasing trend in the years 2010–2014 when they grew from 6.2 to 9.8 per 1000 patients. Despite the similar highest values observed from 2014–2017, they returned to the output values of 6.5–6.3 per 1000 patients in 2018 and the following two years.

Detailed analysis of the changes in the number of examinations ordered by the hospital departments in 2019 and 2020 showed the general increase in the number of CTs (Table 3). This was especially noticeable in the case of specialized COVID-19 units (Stroke and Internal Medicine 1 Departments). The number of US examinations decreased significantly in these departments as a result of patient isolation and the tightening of the recommendations to perform the US according to radiological societies. Other departments showed an increase in US examinations, in particular the Surgery Department. The downward trend in the number of X-ray examinations concerned most of the departments with the exception of Surgery, which, as in the case of the US, showed a significant increase.

## 4. Discussion

Our current results concerning the rapidly increasing annual quantity of diagnostic imaging examinations are in line with previously reported findings since the 1990s [1,2]. These observations prove a constant, near two-fold increase in the number of CT and US examinations through the subsequently analyzed decades.

The decreasing number of X-ray scans, constituting an exception, is probably due to the tendency of replacing radiography with CT. The examination brings more detailed information about the structure and function of organs than X-ray, and thus becomes the preferred diagnostic modality in the standards of management of care, e.g., in urology or pulmonology The 1.8 fold increase in CT examinations between 2010 and 2020 may also be due to the fact that modern helical CT scanners enable faster examination, thus, more examinations can be performed in one day [24]. An undesirable side effect of this, and the fact that CT is finding more applications, is the higher dose of radiation to which patients are exposed. This applies in particular to hospitalized patients who more often require multiple CT scans. We did not find an unequivocal reason to explain the increasing linear trend in the number of bedside X-ray examinations, nor the near-constant number of interventional procedures per 1000 patients. One possible explanation for the increasing number of bedside X-rays was the increase in ICU beds in 2015, as this department commissioned the vast majority of these examinations during the study period that constituted 73.3% and 52.5% of all bedside X-rays performed in 2019 and 2020, respectively (Table 3). The number of interventional procedures depended on the schedule of admissions at wards that referred patients to elective interventional procedures, as well as the availability of a secured place in the ICU, the number of interventional on-call duties, and the number of working interventional radiologists. These factors had not changed significantly, therefore the number of digital subtraction angiography (DSA), thrombectomy, and aneurysm embolization procedures remained at a similar level (per 1000 patients) during the last decade, despite slight fluctuations. The 1.7 fold increase in US quantity resulted mainly from the lack of contraindications for this diagnostic method and the growth of both the popularity and the availability of US.

The introduction of rigorous methods of preventing the spread of coronavirus-2 and the public’s fear of infection negatively affected many groups of patients, including those treated for oncological, cardiological, and other causes [25,26,27]. Limited access to primary healthcare meant that patients delayed or canceled their routine medical visits and had to seek help in emergency departments. Among all patients admitted to the hospital in 2020, the percentage of those in a serious condition, and thus requiring more extensive diagnostics, was higher than in 2019. This seems to be confirmed by the increase in the number of CT, US, and all bedside X-ray examinations as well as bedside X-rays performed outside the ICU department (Figure 2 and Table 3). During the pandemic in 2020, despite the much smaller number of patients treated that year, there was a further increase in the number of CT and US examinations. This was significantly influenced by the new COVID-19 lung imaging recommendations which covered all radiological modalities, in particular the CT. We observed a near 5 fold increase in the number of any CT examinations that included the chest area in 2020 compared to the previous year, mainly due to the need to assess the severity of the disease and the response to treatment, as well as the follow-up with COVID-19 patients. The increase in the number of US and bedside X-ray examinations also resulted from more frequent lung imaging and diagnosing the extra-pulmonary COVID-19 symptoms (Table 3).

The Organization for Economic Co-operation and Development (OECD) analyses provide further insights into the pace of the increase in imaging examinations worldwide. In Poland, the number of CT examinations performed in the years 2014 and 2018 accounted for 65.8 and 85.4 examinations per 1000 inhabitants, respectively [13]. At the same time, the largest number of studies per 1000 inhabitants, according to the latest data from 2018 and 2019, was reported in the USA—278.5 compared to the smallest quantities reported in Finland—57.5 (Europe) and Costa Rica—38.4 (world) in the same years.

Present-day devices used in radiology provide higher spatial and contrast resolution of the visualized tissues. The greater detail of imaging tests allows for more accurate detection of diseases but requires greater accuracy from the radiologist and more time for analysis. The growing demand for imaging examinations, especially in oncology, is an important issue regarding the time that the radiologist must devote to describing the examination. Assessing disease response to treatment requires a comparison of the current and the previous studies, which in practice means re-evaluating several images at the same time.

Although Poland has the smallest number of doctors per 100,000 inhabitants in the EU, their numbers are slowly increasing. According to Statistics Poland (GUS), the last decade saw an increase in the number of practicing physicians in Poland from 42.2 per 10,000 inhabitants in 2010 to 58.8 in 2019 [28]. The reports issued by the Polish Chamber of Physicians and Dentists (NIL) show that in 2020 there were 4199 radiologists in Poland, including 3857 currently practicing; a number that has undergone a major change since 2010 (3355 and 2904, respectively) [29]. Furthermore, as of 31 May 2021, there are 4271 radiologists in Poland, including 3928 currently practicing. It seems, however, that the growing number of doctors does not meet the needs of the increasing workload in the diagnostic imaging sector. The 32% increase in the number of practicing radiologists in Poland during the last decade is barely a significant part of the increase in the number of CT and US examinations observed in our study during this time (78% and 66% respectively). On top of that, no significant changes in the number of personnel occurred in the Department of Radiodiagnostics, Interventional Radiology and Nuclear Medicine in the analyzed period, which translates into an even greater, local disproportion. Of note, radiology is among specialties with the highest physician task load (PTL) scores [14,15].

A possible way of reducing the workload and expenditure on diagnostic imaging would be to decrease the number of costly and inadequate imaging studies, especially in the presence of the reports highlighting that, in some cases, as much as 47% of patients receive unnecessary diagnostic imaging examinations [10,11,30]. It could, however, be wishful thinking. We believe that patients are becoming more conscious and demanding while their awareness of the potential risk of X-ray radiation exposure is low [31,32]. Meanwhile, physicians who fear the consequences of making a medical error, knowing that imaging greatly facilitates the diagnostic process, may be ordering more imaging examinations inconsiderately.

What could prove to be a milestone in adapting radiology to future healthcare expectations are computer programs and applications that automatically analyze the imaging examinations for the presence of pathologies. These programs, designed on the principle of machine learning, can search, among others, for lung nodules, ischemic changes in the brain, or lesions characteristic of COVID-19 in chest CT and X-ray examinations [33,34,35,36,37].

The discrepancies between results of a single and double reading of imaging studies by radiologists can differ by as much as 22%, indicating a significant advantage of the latter method [38]. Double reading, however, requires additional resources such as staff, time, and money that are lacking in the current healthcare systems.

Studies analyzing the efficacy of computer-aided detection (CAD) support the opinion that these programs can facilitate radiological diagnosis by increasing its accuracy when a single reading is compared to a double reading assisted by CAD [39,40,41].

Artificial intelligence (AI) has also contributed to the detection of lung changes characteristic of COVID during the pandemic [19,20,42,43]. CAD programs can define the presence of SARS-CoV-2 infection and evaluate the severeness of the disease by analyzing the characteristic opacity patterns and their volume inside the lungs both in CT and X-ray images and, thus, help doctors to diagnose and properly treat COVID-19 patients. Nevertheless, some critical reviews suggest that CAD programs are far from perfect and may cause misleading conclusions [35]. These mixed opinions prove that CAD performance in medicine, however helpful in many cases, is dependent on a human–professional and further work on the AI is needed to achieve satisfactory results. However, they will undeniably constitute an important part of future radiology and may become a factor that will increase the efficiency of radiology departments and improve the quality of work of radiologists.

## 5. Conclusions

During the last decade, the number of CT and US examinations nearly doubled, continuing the trend that has been observed since the mid-1990s. The SARS-CoV-2 pandemic has not changed this trend despite the much smaller number of patients treated that year, mainly due to the new lung imaging recommendations. The increase in the number of radiologists has not kept pace with the demand for diagnostic imaging examinations, leading to an increase in the workload and possible overburden. The rapidly increasing number of radiological examinations results in an increase in healthcare expenditure and greater population exposure to radiation. There is a need for new computer programs and applications that will aid radiologists with the growing workload disproportion by reducing the time necessary to analyze imaging examinations and ensuring the reliability of the results, as well as potentially minimizing the related costs.

In conclusion, the answer to the question “are we overdoing it?” seems to be affirmative. However, resolving this complex problem would require major changes to the healthcare system, including, among others, higher resource awareness, more resource investment, and technology development.

## Figures and Tables

**Table 1:** The sum of the different types of examinations per 1000 patients performed yearly during 2010–2020. The years are presented in descending order.

Year	X-Ray	Bedside X-Ray	US	CT	Interventional Radiology	Total Number of Examinations	Total Number of Patients *
2020	96.9	16.4	86.5	155.7	6.3	18,929	52,316
2019	99.8	13.7	81.2	139.8	6.4	22,446	65,858
2018	95.8	15.9	77.2	112.1	6.5	19,536	63,536
2017	105.6	17	88.3	110.1	9.9	19,507	58,960
2016	98.4	15.1	68	111.1	8.6	18,165	60,316
2015	89.8	11.7	57.5	104.2	9.8	16,714	61,232
2014	89.3	12.6	58.6	111.9	9.8	16,869	59,794
2013	96.8	10.5	55.8	105.6	8.4	16,077	58,031
2012	99.2	8.2	56	113	9.2	17,038	59,641
2011	107.8	3.1	56.6	105.7	7.9	17,082	60,763
2010	115.1	3.1	52.1	87.4	6.2	17,834	67,555

* The total number of patients in the emergency room, wards, and clinics (patient transfer from the emergency room to the ward and the two units ordering the same examination were omitted). CT—computed tomography, US—ultrasound.

**Table 2:** The number of chest CT examinations per 1000 patients performed in 2019 and during the COVID-19 pandemic in 2020. The years are presented in descending order.

Year	HRCT	All Chest CTs	All CTs That Included the Chest Area	*p*
2020	8.7	28.6	52.3	<0.001
2019	2.0	9.5	10.8	

CT—computed tomography, HRCT—high resolution computed tomography.

**Table 3:** The number of examinations per 1000 patients performed in 2019 and 2020 in individual hospital departments. The detailed list includes departments ordering the largest number of examinations. Other departments, as well as hospital and external clinics, are presented together.

Department	CT		US		X-Ray		CT	US	X-Ray
	2020	2019	2020	2019	2020	2019	2020 vs 2019		
ER	444	388	211	185	191	192	1.14	1.14	0.99
ICU	1246	1087	554	775	1341 *	1614 *	1.15	0.71	0.83 *
Surgery	175	156	263	197	394	245	1.12	1.34	1.61
Internal Medicine 1	349	262	82	113	426	569	1.33	0.73	0.75
Internal Medicine 2	447	323	759	1235	792	849	1.38	0.61	0.93
Gastro-enterology	117	121	59	58	178	189	0.97	1.02	0.94
Neurology	147	127	265	218	222	197	1.16	1.22	1.13
Stroke	752	543	987	1071	646	643	1.38	0.92	1.00
Other **	159	161	57	46	70	84	0.99	1.24	0.83

* Bedside X-ray, ** Other hospital departments (including Allergology, Gynecology, Neonatology, Neurological Rehabilitation, Neurosurgery), hospital and external clinics. CT—computed tomography, ER—emergency department, ICU—intensive care unit, US—ultrasound.

## Data Availability

The data presented in this study are available on request from the corresponding author.
